# The American Dental Society of Japan

**Published:** 1899-09

**Authors:** 


### THE AMERICAN DENTAL SOCIETY OF JAPAN.

THIS title reads strangely in view of the comparatively short time since Japan was opened to the civilization of the outside world, but it is an accomplished fact, as this body was organized in Tokio, Japan, on June 3, 1899, with its constitution and by-laws and a partial list of officers.

Our old friend and co-worker in dental education, Dr. Louis Ottofy, seems to have been the active spirit and has been made its first president.

The time will come, doubtless, when Japan will follow in the path of other civilizations, and bar out all American dentists, but for the present the American Dental Society of Japan may be congratulated as the first organized effort to transplant American ideas of dentistry in the Orient.

